# Degradation of Pesticides Using Semiconducting and Tetrapyrrolic Macrocyclic Photocatalysts—A Concise Review

**DOI:** 10.3390/molecules28227677

**Published:** 2023-11-20

**Authors:** Giusi Piccirillo, Rodrigo B. De Sousa, Lucas D. Dias, Mário J. F. Calvete

**Affiliations:** 1 Coimbra Chemistry Centre-IMS, Department of Chemistry, University of Coimbra, Rua Larga, 3004-535 Coimbra, Portugal; giupiccirillo12@gmail.com; 2 Laboratório de Novos Materiais, Universidade Evangélica de Goiás, Anápolis 75083-515, GO, Brazil; rod.borges.qi@gmail.com

**Keywords:** photodegradation, environment, wastewater, pesticides, green processes

## Abstract

Exposure to pesticides is inevitable in modern times, and their environmental presence is strongly associated to the development of various malignancies. This challenge has prompted an increased interest in finding more sustainable ways of degrading pesticides. Advanced oxidation processes in particular appear as highly advantageous, due to their ability of selectively removing chemical entities form wastewaters. This review provides a concise introduction to the mechanisms of photochemical advanced oxidation processes with an objective perspective, followed by a succinct literature review on the photodegradation of pesticides utilizing metal oxide-based semiconductors as photosensitizing catalysts. The selection of reports discussed here is based on relevance and impact, which are recognized globally, ensuring rigorous scrutiny. Finally, this literature review explores the use of tetrapyrrolic macrocyclic photosensitizers in pesticide photodegradation, analyzing their benefits and limitations and providing insights into future directions.

## 1. Introduction

Agricultural chemicals, known as agrochemicals, encompass fertilizers and pesticides. The latter consist of insecticides, herbicides, fungicides, and nematicides. The extensive use of intensive agricultural practices worldwide has led to a significant rise in the numbers and types of agrochemicals present in continental and marine waters. This trend is attributable to the growth of the agrochemical industry. Exposure to pesticides is inevitable, and studies have established a definite connection between pesticide exposure and the emergence of cancer in both adults and children [1,2]. Individuals who are closely linked with pesticide exposure are at an elevated risk of various malignancies, including those that are cancer-related, neurological disorders, and endocrine disruptions [3,4]. Furthermore, studies have investigated the occurrence and fate of pesticides in the environment, and their long-term presence is significantly associated with detrimental effects on ecosystems [5,6,7,8,9,10].

Therefore, there is an urgent need to develop methods to destroy/remove these pollutants in the environment. Pesticides can be removed by two main methodologies: non-destructive and destructive. The former includes physical methods such as adsorption, liquid extraction, and membrane separations. The aspects related with adsorption strategies for pesticide removal have been very well reviewed in recent years, with comparisons in the efficacy of these treatment processes with destructive methods [11,12,13,14]; for that reason, they are not the focus of this review.

Physicochemical methods have several drawbacks, with the most significant being sludge formation, while destructive methods, such as chemical oxidation and biodegradation techniques, may offer a destruction process with less residual outcomes. The latter strategy has been widely followed and extensively reviewed in recent years [14,15,16], representing a very interesting approach from a sustainable point of view, but can be hampered by the often recalcitrant nature of wastewater pollutants [17]. Therefore, chemistry-based techniques have evolved and are currently regarded as effective alternatives for wastewater treatment. Of these, chlorine gas/hypochlorite oxidations are traditional disinfection methods for the degradation/removal of contaminants [18]. However, they can also lead to the formation of halogenated species that are potentially carcinogenic. Advanced oxidation processes (AOPs) have become the most favorable methods for oxidizing and degrading pollutants, and they consist of both photochemical and chemical processes.

### 1.1. Advanced Oxidation Processes (AOPs) for the Degradation of Pesticides

**Figure 1:**
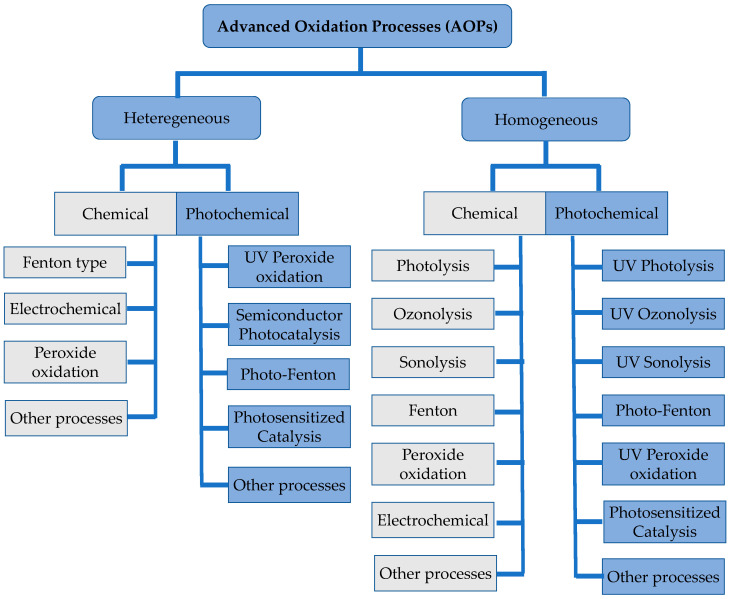
Advanced oxidation processes (AOPs) subcategories.

AOPs (Figure 1) are based on the generation of highly reactive and rather unselective species, such as hydroxyl (HO^•^) or sulfate SO_4_^•−^ radicals, or singlet oxygen (^1^O_2_) [19,20,21]. In this review, we focus solely on photochemical processes, particularly highlighting the use of photosensitizing catalysts in the degradation of pesticides. Mechanistic and reaction pathways have been adequately discussed in previous literature [22,23,24,25,26] and are not the main focus of this review; however, we provide a brief introduction with references to mechanistic considerations before reviewing and discussing multiple pivotal works that report several important photochemical approaches. Finally, we will analyze the literature regarding the application of tetrapyrrolic macrocycle photosensitizers on the photodegradation of pesticides.

### 1.2. Photooxidation Mechanisms

#### 1.2.1. Mechanisms Using Semiconductors as Catalysts

**Figure 2:**
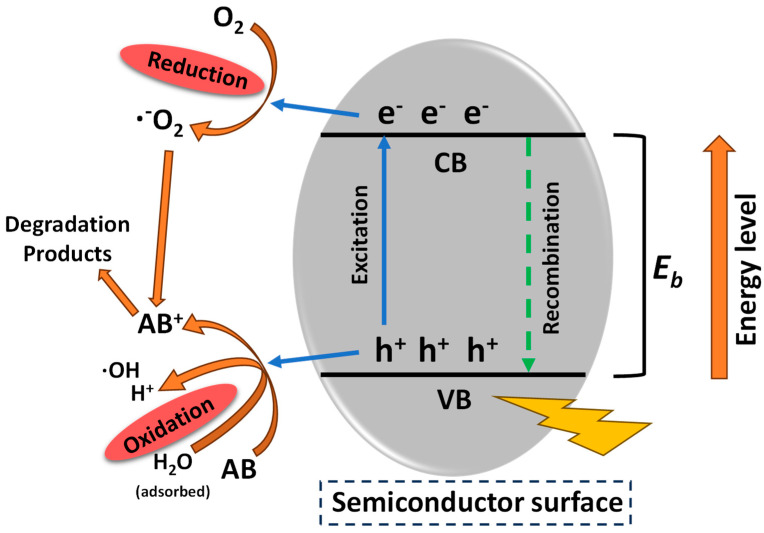
General mechanism for reactive oxygen species (ROS) formation using semiconductors.

Semiconductors are materials classified as both conducting and insulating [27]. Metal oxides, metal ferrites, and metal sulfides are examples of semiconductors, which have particular band gap energies. Semiconductor nanomaterials have been widely used because of their ability to harness light energy and produce charged carriers [26,28,29,30]. The typical mechanism involving semiconductors is depicted in Figure 2.

When a semiconductor photocatalyst absorbs light with energy equal to or greater than its band gap energy, electron-hole pairs are formed. These pairs split into positively charged holes (h^+^) in the valence band (VB) and electrons (e^−^) in the conduction band (CB) (Equation (1)). Positively charged holes can promote oxidation reactions of organic molecules that are adsorbed on the surface of the catalyst, or they can generate ROS via the conversion of adsorbed water or hydroxide ions to HO^•^ (Equation (2)). At the same time, electrons in the CB can be transferred (reduction) to molecular oxygen, producing O_2_^•−^, which is subsequently converted into H_2_O_2_, or promote the reduction of H_2_O_2_ to HO^•^ (Equations (3)–(6)). The hydrogen peroxide also acts as an electron receptor, generating extra hydroxyl radicals (Equation (7)), and the adsorbed substrate pesticide (PEST_ads_) may also be directly oxidized by electron transfer (Equation (8)) [31,32].

(1) $$\mathrm{SC}\;\overset{h\upsilon }{\to }\mathrm{PS}(e^{-}+h^{+})$$

SC_surf_(h^+^) + H_2_O_ads_ + HO^−^ → SC_surf_ + 2 HO^•^_ads_ + H^+^ (2)

SC(e^−^) + O_2_ → SC + O_2_^•−^ (3)

O_2_^•−^ + H_2_O ↔ HO_2_^•^ + HO^−^ (4)

2 HO_2_^•^ → H_2_O_2_ + O_2_ (5)

O_2_^•−^ + H_2_O_2_ → O_2_ + HO^•^ + HO^−^ (6)

SC_surf_(e^−^) + H_2_O_2_ → SC_surf_ + HO^−^ + HO^•^ (7)

SC(h^+^) + PEST_ads_ → SC + PEST_ads_^+^ (8)

#### 1.2.2. Mechanisms Using Photo-Fenton Catalysts

**Figure 3:**
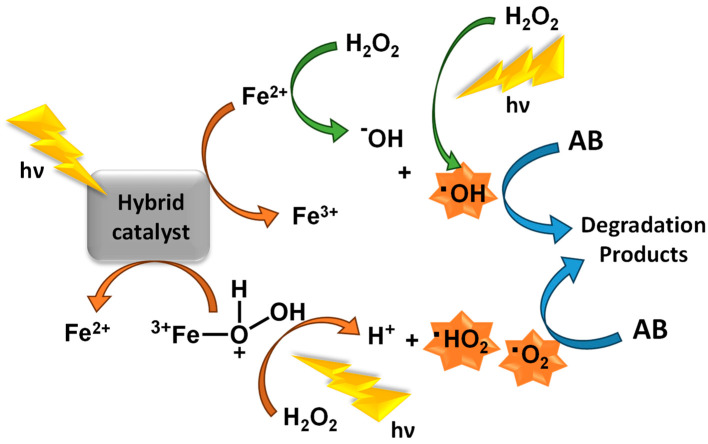
General mechanism for ROS formation using photo-Fenton catalysts.

Henry Fenton developed an aqueous solution of hydrogen peroxide and iron ions to oxidize organic compounds in acidic media, capable of generating hydroxyl radicals, postulated as Fenton’s reagent [33]. It was not until almost a century later that it was first used to degrade pollutants [34], triggering the development of this process, which has been applied with success in the degradation of many pollutants [35,36]. The typical mechanism involving photo-Fenton catalysts is depicted in Figure 3.

Several reactions occur during the process [37] but, mostly, there is the reaction responsible for producing HO^•^ radicals from hydrogen peroxide and oxidizing Fe^2+^ to Fe^3+^ (Equation (9)), along with the formation of the Fe(HO)^2+^ species (Equation (10). The efficiency of this oxidation process can be increased by combining it with light irradiation, known as the photo-Fenton process [38,39]. This enhancement is possible mainly due to the production of additional hydroxyl radicals through the photolysis of Fe(HO)^2+^ complexes (Equation (11)), via direct H_2_O_2_ photolysis (Equation (12)) or photo-reduction of ferric ions Fe^3+^ (Equation (13)).

Fe^2+^ + H_2_O_2_ → Fe^3+^ + HO^−^ + HO^•^ (9)

Fe^3+^ + HO^−^ → Fe(HO)^2+^ (10)

(11) $${\mathrm{Fe}(\mathrm{HO})}^{2+}\;\overset{h\upsilon }{\to }{\mathrm{Fe}}^{2+}+{\mathrm{HO}}^{•}$$

(12) $$H_{2}O_{2}\;\overset{h\upsilon }{\to }2{\mathrm{HO}}^{•}$$

(13) $${\mathrm{Fe}}^{3+}+H_{2}O_{2}\;\overset{h\upsilon }{\to }{\mathrm{Fe}}^{2+}+{\mathrm{HO}}^{•}\;H^{+}$$

The efficiency of these processes is primarily influenced by pH, temperature, catalyst, hydrogen peroxide, and organic substrate concentration. The pH value appears to be the most critical variable in Fenton and photo-Fenton process performance. In fact, radical quenching may take place, being necessary to perform the reactions within a narrow pH range, around pH = 3, where Fe(HO)^2+^ species are prevalent [40]. In addition, the usage of peroxymonosulfate (PMS) as an oxidant can trigger a process similar to photo-Fenton, generating sulfate radicals, which are also commonly used in several AOPs [41,42]. Radiation, such as UV, can efficiently activate PMS. Two activation pathways can occur when radiation is used: the first is the cleavage of the O-O bond induced by the energy input (Equations (14) and (15)). In the second one, the radiation could dissociate water molecules (Equation (16)), producing the electron that activates PMS by electron conduction (Equations (17) and (18)).

(14) $${S_{2}O_{8}}^{2-}\;\overset{h\upsilon }{\to }2\;{{\mathrm{SO}}_{4}}^{•-}$$

(15) $${{\mathrm{HSO}}_{5}}^{-}\;\overset{h\upsilon }{\to }{{\mathrm{SO}}_{4}}^{•-}+{\mathrm{HO}}^{•}$$

(16) $$H_{2}O\;\overset{h\upsilon }{\to }H^{•}+{\mathrm{HO}}^{•}$$

S_2_O_8_^2−^ + H^•^ → SO_4_^•−^ + SO_4_^2−^ + H^+^ (17)

HSO_5_^−^ + H^+^ → SO_4_^•−^ + H_2_O (18)

#### 1.2.3. Mechanisms Using Tetrapyrrolic Macrocyclic Photosensitizers

**Figure 4:**
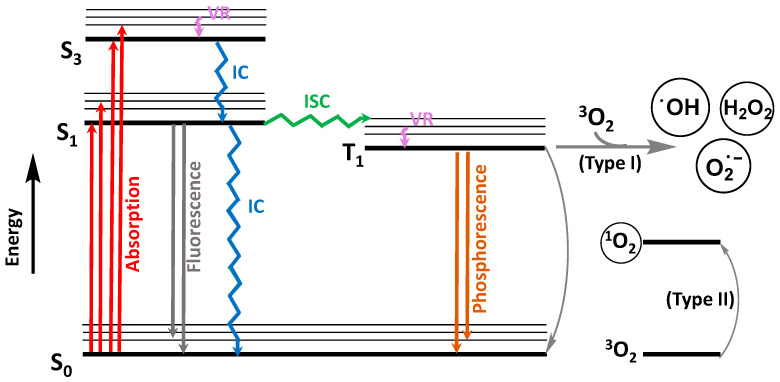
Adapted Jablonski [59] diagram depicting the general mechanism for ROS formation using organic photosensitizers.

Photosensitizers are light-absorbing agents that modify the course of a photochemical reaction. They usually serve as catalysts that, ideally, are not consumed during the photochemical reaction. Among the photosensitizer family, which can either be of inorganic (e.g., semiconductors) or organic origin, the tetrapyrrolic macrocycles are exceptional photosensitizing agents that have been used in a vast array of photonic applications [43,44,45,46,47,48,49,50,51,52,53,54,55,56,57,58]. The typical photochemical mechanism of oxidation of any molecule (e.g., pesticides) using photosensitizing catalysts, such as tetrapyrrolic macrocycles, is shown in Figure 4.

The adapted Jablonski [59] diagram in Figure 4 illustrates the relevant electronic transitions that promote ROS formation in photocatalysis, causing intramolecular bond ruptures and ultimately leading to the desired mineralization and degradation of the target molecules. Briefly, the excitation of the photosensitizer from the singlet ground state, S_0_, to a singlet excited state (S_1_, S_2_,…, S_n_) is promoted by the absorption of light of suitable wavelengths. Typically, a transition to a higher-order singlet excited state (S_2_–S_n_) is followed by a rapid transition to S_1_ via non-radiative vibrational relaxation (VR) and internal conversion (IC). Singlet excited states are transient and can directly return to the ground state by fluorescence emission or IC. Otherwise, intersystem crossing (ISC) to a triplet-excited state (usually T_1_) may occur. This state has a longer lifetime and can interact with oxygen by two mechanisms: (i) direct or indirect electron transfer to molecular oxygen can occur (Type I mechanism), eventually leading to the formation of HO^•^ and/or O_2_^•−^ and/or H_2_O_2_; (ii) energy transfer can occur between the catalyst in T_1_ and ^3^O_2_ (Type II mechanism), causing the concomitant return of the catalyst to the ground state (S_0_) and the formation of singlet oxygen (^1^O_2_) [48,49,60].

In type I reactions [61], the photosensitizer (PS), when in the triplet excited state (^3^PS*), can react directly with substrates (SUB) present in the medium (typically aqueous) to generate either cationic or anionic radical species (PS^•+^ and/or PS^•−^; Equations (19) and (20)). In particular, the latter species (PS^•−^) can interact with molecular oxygen dissolved in the medium, producing superoxide anion (O_2_^•−^), while returning to its ground state (Equation (21)). Subsequently, the reversible protonation of O_2_^•−^ can result in the formation of peroxo radicals (HOO^•^; Equation (22)), which may further combine to form H_2_O_2_ (Equation (23)). Lastly, the reaction of H_2_O_2_ with O_2_^•−^ can lead to the formation of HO^•^ (Equation (24)), which is recognized as the most reactive ROS due to its higher one-electron redox potential, thus granting the capacity to oxidize a broad range of substrates [60].

^3^PS* + SUB → PS^•+^ + SUB^•−^ (19)

^3^PS* + SUB → PS^•−^ + SUB^•+^ (20)

PS^•−^ + O_2_ → PS + O_2_^•−^ (21)

O_2_^•−^ + H_2_O ↔ HOO^•^ + HO^−^ (22)

2 HOO^•^ → H_2_O_2_ + O_2_ (23)

O_2_^•−^ + H_2_O_2_ → O_2_ + HO^•^ + HO^−^ (24)

These oxidation mechanisms can occur either separately or in combination between each other, and are complementary to the ROS formation pathways, which act as driving forces for pollutant degradation, in particular for pesticides, which is the focus of our review.

The review proceeds with a brief general introduction to the mechanisms of photochemical advanced oxidation processes (AOPs). This is followed by a concise literature review on the photodegradation of pesticides using metal oxide-based semiconductors as photosensitizing catalysts. The reports discussed here have been selected based on their relevance and impact, endorsed by general worldwide recognition. Finally, a literature review on the use of tetrapyrrolic macrocyclic photosensitizers in the photodegradation of pesticides is given, discussing the advantages/drawbacks of their use and presenting a perspective on future directions.

## 2. Photodegradation of Pesticides

Several categories of pesticide contaminants, including organophosphates, azotic heterocycles, organo-chlorinated and phenol compounds, neonicotinoids, natural derived compounds, and others, are now on every governing body’s watchlist [62,63] (refer to Table 1 for a list of the pesticides discussed in this review’s photodegradation section). While the list in Table 1 is far from comprehensive, in the literature, more comprehensive information on pesticide chemical categories can be found [64,65,66].

### 2.1. Using Unitary Metal Oxide Semiconductors

Semiconductor photocatalysis is an indirect photolytic advanced oxidation process (AOP) which was first applied for the photo-induced splitting of water on TiO_2_ electrodes [67]. This method has since been utilized in a variety of redox reactions involving both organic and inorganic substrates [68]. Currently, TiO_2_ remains the most commonly reported and utilized photocatalytic material for degrading general pollutants in UV light aqueous environments, according to academic research. The primary benefits of using TiO_2_ as a semiconductor are its relatively low cost, non-toxic nature, and high photocatalytic activity [69,70]. However, the main disadvantage lies in its restricted photocatalytic efficiency under solar irradiation as the UV light content in the solar spectrum only comprises about 3–6% [71]. Efforts have been made to overcome this issue, including the use of different metal oxides or related sulfides, attempting to take advantage of more favorable absorption features [72]. Table 2 summarizes a critical selection of pivotal works reported in the literature, on processes and reaction conditions to promote the photodegradation of pesticides using single metal oxide semiconductors as photocatalysts.

Pelizzetti’s group was the first to use semiconductor photocatalysts in the degradation of pesticides [73,80], specifically pentachlorophenol (PCP) [73]. The widespread use of **TiO_2_** as a photocatalyst may have stemmed from these these works, which reported that, under solar simulator irradiation, full degradation of PCP (12 mg L^−1^) was observed after 1.5 h in the presence of **TiO_2_** (2 g L^−1^), faster than in comparison with other semiconductors, such as **ZnO** (ca 2 h), or **CdS**, **WO_3_**, and **SnO_2_**, which only achieved incomplete degradation rates even after 3 h (Table 2, Entry 1). The same group conducted a subsequent study, comparing the effectiveness of **TiO_2_ P-25** vs. **TiO_2_** made hydrophobic by surface modification **TiO_2_** (**P-25**) powder with octyltrimethoxyl silane (**T-805**) [74]. The results indicated that the modified **T-805** photocatalyst was superior to **TiO_2_ P-25** in a *n*-hexane-aqueous medium, achieving the degradation of permethrin (PER) even at a high concentration of 17 g L^−1^ (90% deg. under UV light, after 20 h; 80% deg. under solar sunlight, after 10 h) (Table 2, Entry 2). A first order reaction rate of k = 0.002 min^−1^ was determined, attributing HO^•^ and O_2_^•−^ as the main ROS species capable of degrading the pesticide. The photodegradation process involved the dechlorination of the substrate, measured through mineralization to Cl^−^ ions and to CO_2_, which was found to reach 5% after 24 irradiations.

**Figure 5:**
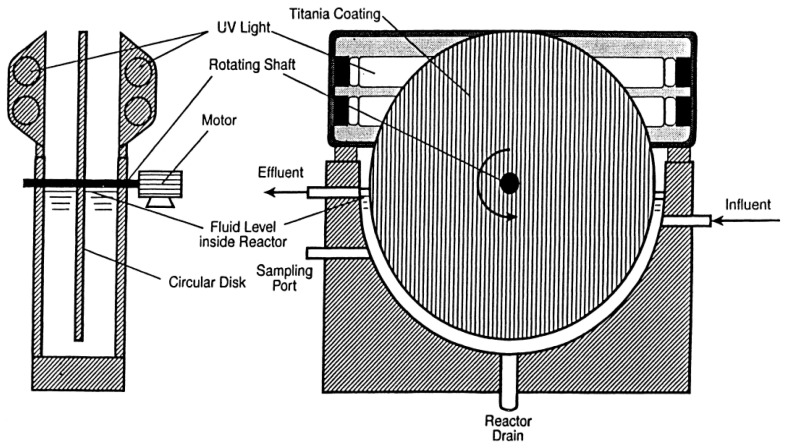
Schematic representation of the rotating disk photocatalytic reactor used by Suidan. Adapted with permission from ref. [75]. Copyright 2000 Elsevier B.V., Amsterdam, The Netherlands.

In a larger study, Suidan [75] reported the photocatalytic degradation of pesticides containing chlorine atoms, specifically chlorinated phenols, and lindane. These were assessed in a continuous flow **TiO_2_** rotating disk photocatalytic reactor, which is shown in Figure 5.

Under UV light irradiation, all chlorophenols, including 4-chlorophenol (4-CP), 2,4-dichlorophenol (2,4-DCP), all trichlorophenol isomers (2,3,5-TCP, 2,4,6-TCP, and 2,3,6-TCP), and pentachlorophenol (PCP) were photodegraded in aqueous medium, while lindane (LIN) was degraded in acetone (Table 2, Entry 3). Degradation levels above 90% were achieved for CP, 2,4-DCP, 2,3,5-TCP, and PCP, while 2,4,6-TCP and 2,3,6-TCP reached 86–89%. Pseudo-first-order rates were determined, resulting in the following trend: k_1_ = 0.090 min^−1^ (2,3,5-TCP) > 0.067 min^−1^ (2,4-DCP) > 0.032 min^−1^ (4-CP) > 0.017 min^−1^ (2,4,6-TCP) > 0.025 min^−1^ (PCP) > 0.020 min^−1^ (2,3,6-TCP) > 0.005 min^−1^ (LIN). Furthermore, the authors suggested OH^•^ as the main mobile radical responsible for the degradation of the pesticides.

Thampi [76] also reported the photodegradation of atrazine (ATR) using suspended and supported **TiO_2_**. When a 90 mW cm^−2^ intensity lamp was used for irradiation, with λ centered at 290 nm, a ~90% degradation was observed after 60 min (Table 2, Entry 4). However, reducing the light intensity to 50 mW cm^−2^ caused a reduction in efficiency. This efficiency was attributed to the formation of HO^•^ and O_2_^•−^ radicals, resulting in a 90% reduction in the dissolved organic carbon (DOC) present after 90 min.

Saien [77] also promoted the photodegradation of carbendazim (CAR) using a **TiO_2_** photocatalyst with 250 W Hg lamp irradiation. Degradation reached 90% in 90 min, with a rate constant of k_1_ = 0.030 min^−1^. The authors attributed the main degrading ability to HO^•^ radicals (Table 2, Entry 5).

**Figure 6:**
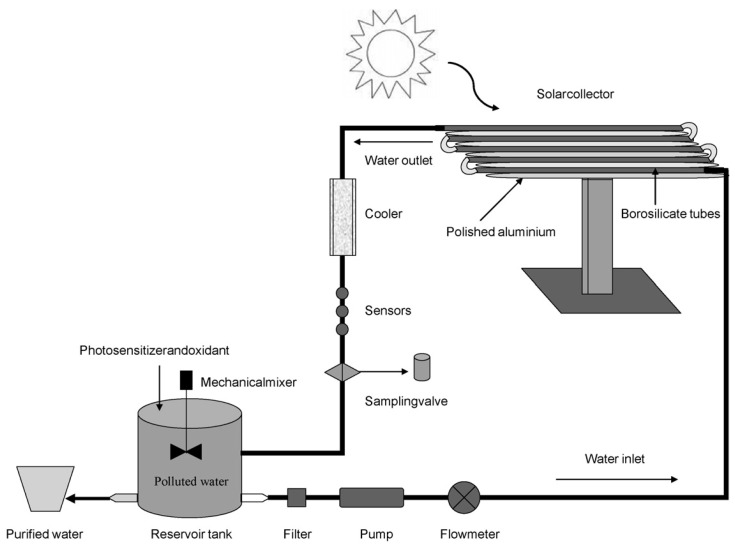
Scheme of the pilot plant used for the photocatalytic experiments by Navarro. Adapted with permission from ref. [78]. Copyright 2009 Elsevier B.V. Amsterdam, The Netherlands.

Navarro [78] used **ZnO** as a semiconductor photocatalyst instead of **TiO_2_** in the degradation of several pesticides, including azoxyxtrobin (AZO), kresoxim-methyl (KRM), hexaconazole (HEX), tebuconazole (TEB), triadimenol (TRI), pyrimethanil (PYRI), primicarb (PRI), and propyzamide (PRI) (Table 2, Entry 6). Photocatalytic experiments were performed in a pilot scale under natural sunlight irradiation (Figure 6). The results showed the following decreasing first-order kinetics: k = 1.4727, 0.7370, 0.7019, 0.6014, 0.5862, 0.5475, 0.4762, and 0.3885 min^−1^ for PRI, PYRI, PRO, AZO, KRM, TEB, HEX, and TRI, respectively. The addition of Na_2_S_2_O_8_ resulted in a significant reduction in reaction time, demonstrating a faster reaction time than **ZnO** alone. Complete degradation occurred only after one to two hours, with the primary responsible ROS being HO^•^ and SO_4_^•−^.

Another example was given by Madras [79], who prepared CuS semiconductors using copper precursor in the presence of sodium thiosulfate (**CuS-ST**), thioacetamide (**CuS-TA**), and thiourea (**CuS-TU**). These were twisted in the photodegradation of 4-chlorophenol (4-CP) under irradiation with a 400 W metal halide lamp. The best performance was achieved by **CuS-TU**, reaching 62% degradation after 5 h irradiation against 40% and 25% using **CuS-TA** and **CuS-ST**, respectively. Catalyst **CuS-TU** exhibited the highest pseudo-first-order rate constants k = 0.003 min^−1^ vs. 0.002 and 0.0017 min^−1^ for **CuS-TA** and **CuS-ST**, respectively. Moreover, there was a 40% TOC decrease after five hours irradiation.

### 2.2. Using Hybrid Photocatalysts

Many efforts have been devoted to enhancing the performance of semiconductor-based photocatalysts for practical applications. The initial switch to the use of different metals evolved to their modification and/or combination of organic and/or inorganic counterparts [81,82], aiming to achieve longer wavelength absorption and prevent recombination of charge carriers [83,84]. Table 3 summarizes a critical selection of pivotal works reported in the literature, on processes and reaction conditions to promote the photodegradation of pesticides using hybrid (binary and ternary) photocatalysts.

Fenoll [85] conducted a study on the aqueous photocatalytic degradation of chlorantraniliprole (CAP) by using suspensions of **TiO_2_**, **ZnO,** and **Zn_2_TiO_4_** oxides under light irradiation with 8 W medium pressure Hg lamps. The authors observed that the addition of Na_2_S_2_O_8_ (250 mg L^−1^), as an electron acceptor to the reaction mixtures, significantly improved the elimination of CAP in the aqueous slurries compared to **ZnO** and **TiO_2_** alone, as well as the metal binary **Zn_2_TiO_4_** semiconductor. The presence of S_2_O_8_^2−^ boosted the production of highly reactive SO_4_^•−^ radicals which, in combination with the HO^•^ radicals, improved the degradation ability of the catalyst. **ZnO** performed very well, showing a first-order kinetics constant of k = 0.013 min^−1^. This value was 1.6 times greater than that of **TiO_2_** (0.008 min^−1^) and 7-fold higher than that of **Zn_2_TiO_4_** (0.002 min^−1^) (Table 3, entry 1). The authors attributed to **ZnO** and **TiO_2_** the higher disappearance rates. When comparing **Zn_2_TiO_4_** to the absorption threshold of **ZnO** and **TiO_2_**, both exhibited a more favorable absorption threshold (413, 400 nm, respectively) than **Zn_2_TiO_4_** (376 nm), depending on the light source used (300–460 nm). The authors identified 7 photoproducts (PPs) and observed a 90% dissolved organic carbon (DOC) reduction for **TiO_2_** and **ZnO**, while only 22% DOC reduction was observed for **Zn_2_TiO_4_**.

To leverage more favorable absorption features, binary and even ternary catalysts that incorporate semiconductors have included the use of different metal oxides or related sulfides. A good example was reported by Mishra and coworkers [86], who prepared a binary photocatalyst composed of **Bi_2_O_2_CO_3_** incorporated into copper sulfide (**CuS**). Additives such as urea (U), potassium chloride (KCl), and hexamethylene tetraamine (HMTA) were also used in the synthesis of **Bi_2_O_2_CO_3_**, providing the materials with varying compositions. These were subsequently mixed with **CuS** precursors (in different *w*/*w* proportions) to yield the desired binary heterojunction semiconductor catalysts. The optimal photocatalyst (**CuS_10_/Bi_2_O_2_CO_3_(U_5_K_1_)**, which was prepared using a 5:1 *w*/*w* ratio of urea to KCl and contained 10% *w*/*w* of CuS, successfully degraded chlorpyrifos (CPF) in 95% after 3 h irradiation, with a pseudo-first-order reaction rate of k = 0.031 min^−1^ (Table 3, entry 2). The authors also conducted a mechanistic study that identified 8 PPs resulting from oxidation, dechlorination, hydrolysis, and dehydroxylation processes. These transformations led to the mineralization of CPF (90% TOC reduction, after 3 h), with HO^•^ and h^+^ serving as main reactive species. It is worth noting that this system maintained its activity, even after 5 cycles of reuse.

In an effort to enhance photon collection, some researchers have synthesized ternary heterojunctions [87,88,89,90] using diverse configurations. Kumar and Stadler [87] combined graphitic carbon nitride obtained from biochar (**g-C_3_N_4_**) with **Bi_2_O_2_CO_3_**, which was further immobilized onto magnetic **CoFe_2_O_4_** nanoparticles to yield a **g-C_3_N_4_/Bi_2_O_2_CO_3_/CoFe_2_O_4_** ternary photocatalyst. The photodegradation of paraquat (PAR) was then assessed, both under an 800 W vis light Xe lamp and natural sunlight, and observed that artificial irradiation was marginally better than sunlight (99% deg. vs. 92% deg. in 90 and 120 min, respectively, for artificial and natural irradiation). This effect was corroborated by the determination of their pseudo-first-order reaction rates, which showed a k = 0.069 min^−1^ under Xe lamp and k = 0.060 min^−1^ under sunlight (Table 3, entry 3). The authors observed that HO^•^ and O_2_^•−^ radicals were responsible for the oxidation of the pesticide, and a TOC reduction of 57% was obtained after 3 h reaction.

**Figure 7:**
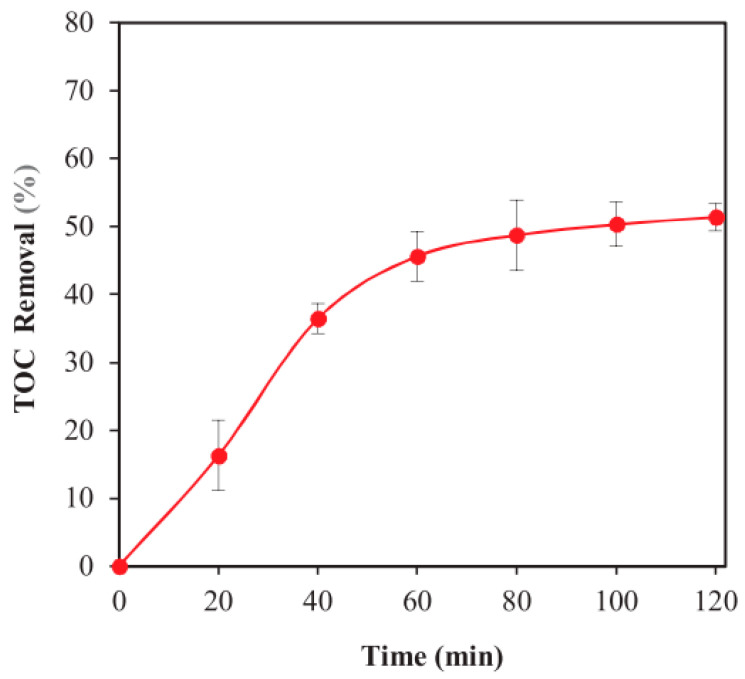
Total organic carbon removal rate during metalaxyl photocatalytic degradation under optimum conditions. Adapted with permission from ref. [89]. Copyright 2019 Elsevier B.V. Amsterdam, The Netherlands.

Zou [88] reported the modification of **BiOI** and **Bi_2_MoO_6_** on graphitic carbon nitride (**g-C_3_N_4_**) for the aqueous degradation of glyphosate (GLY). The efficacy of the method was measured by observing a 94% degradation rate of GLY after 90 min of irradiation, using a tungsten halogen lamp with undisclosed power and spectral range. A reaction rate of k = 0.018 min^−1^ was observed, along with an 83% TOC reduction, after 4.5 h (Table 3, entry 4). The authors proposed a mechanism in which HO^•^ and O_2_^•−^ and h^+^ served as oxidizing species and evaluated the reutilization of the catalyst, which could be reused 5 times with small loss of activity. Kalantary [89] used reduced graphene oxide (**rGO**) as a carbon source, aiming to improve charge utilization and avoid recombination effects. The authors used freshly prepared **rGO** and combined it sequentially with magnetic **Fe_3_O_4_** and **ZnO**. The ternary photocatalyst was tested in the degradation of metalaxyl (MET) and, after 2 h irradiation with Vis-LED lamps at λ = 450 nm, 92% deg. MET was achieved. The catalyst was reused for 5 cycles, with minimal activity loss (Table 3, entry 5). A pseudo-first-order reaction rate k = 0.023 min^−1^ was calculated, and HO^•^ and O_2_^•−^ radicals were identified as the primary oxidizing species. Four PPs were detected, and a 51% TOC reduction was achieved after 2 h irradiation (Figure 7), along with the observation of a toxicity reduction by the PPs after degradation of the pesticide.

Bai used a single metal oxide (**Ag_3_PO_4_**) that was incorporated into a ternary nanocomposite, consisting of graphitic carbon nitride (**g-C_3_N_4_**) and **cucurbit[7]uril (Q[7]**). The hybrid photocatalyst was employed in the degradation of pyroquilon (PYR) and imidacloprid (IMI) under irradiation with a 35 W LED lamp (λ unspecified). The results showed that, after 5 h, 94% and 97% deg. PYR and IMI, respectively, were achieved (Table 3, entry 6). As expected, a pseudo-first-order reaction rate was observed (k = 0.009 and 0.012 min^−1^ for PYR and IMI, respectively). However, catalyst stability was found to be relatively low, showing activity drop of 20% after 5 reutilizations in the photodegradation of PYR.

Strategies for implementing photo-Fenton systems have also been developed, including the traditional use of iron salts in homogeneous aqueous conditions. For example, Doong [91] used iron(II) sulfate as a source of Fe^2+^ ions to promote the photocatalytic degradation of several organic pesticides, such as methamidophos (MET), malathion (MAL), diazinon (DIA), phorate (PHO), and *O*-ethyl *O*-(4-nitrophenyl) phenylphosphonothioate (EPN). The experiments were carried out at pH = 7, using UV-Vis irradiation in combination with H_2_O_2_ oxidant (20 mg L^−1^) in the presence of **FeSO_4_** (7.6 mg L^−1^) or zero-valent **Fe^0^**, which produced the ferrous ion Fe^2+^ at the surface, followed by rapid oxidation to Fe^3+^ (830 mg L^−1^–3.3 mg L^−1^ Fe^2+^). Experimental results indicated that using Fe^2+^ salt was marginally more efficient than using **Fe^0^**, with nearly complete degradation of the pesticides being achieved in 150 min, while longer times were required for the UV/H_2_0_2_ system, in absence of iron catalyst. Reaction kinetics were also evaluated, and rates were found to have the order MET > PHO > EPN > MAL > DIA (Table 3, entry 7). The authors attributed the easiest degradability of MET and PHO to their structural characteristics, such as the lower bond energy of the P-S and C-S bonds, while the other pesticides were mainly photodegraded by action of light and catalyst. Furthermore, HO^•^ radicals were considered the main source for degradation.

Employing the same catalyst (iron(II) sulfate), Malato and Jarvis [92] applied the aqueous homogeneous photo-Fenton catalytic system to the degradation of acetamiprid (ACE) pesticide, using two slightly different irradiation systems (one with 3 UVC low pressure lamps (LPL) with λ = ~256 nm and another with LED lamps, emitting at λ = ~254 nm). The first lamp system demonstrated superior results, reaching full degradation in just 7 min at pH = 2.8 in deionized water (DW). Expectedly, the reaction rate was lower at pH = 8.2 (in synthetic wastewater), requiring 20 min to ACE disappearance (LPL system), while the LED system required over 20 min for full degradation (Table 3, entry 8). The authors also found that a 4:1 (*w*/*w*) ratio of Fe^2+^:H_2_O_2_ was necessary, with [Fe^2+^] = 3 mg L^−1^. The efficiency of the system improved with sequential iron dosing. When the [Fe^2+^] = 3 mg L^−1^ was reached at once, the degradation rate came to a stall after 10 min, with 60% ACE degradation after 30 min, while, when [Fe^2+^] was incremented as 1 + 1 + 1 mg L^−1^, the aforementioned full degradation was reached in 20 min. The authors indicated this necessity to ensure a certain amount of iron was always available in the solution for the photo-Fenton reactions at high pH = 8.2. The observed effect was not noticed at pH = 2.8, where the Fenton reaction promptly produced radicals, due to the presence of hydrogen peroxide. However, ferric iron hydrolysis usually results from higher pH, leading to catalyst depletion. To prevent this disadvantage, incremental iron salt addition was used.

Studies were carried out by Evgenidou [93] using various iron salts as Fenton catalysts to assess the photodegradation of dimethoate (DIM) and methyl parathion (MPA) pesticides under irradiation with a 125 W high pressure Hg lamp. The experiments were carried out in pH = 2.9 aqueous homogeneous conditions (10 mg L^−1^ concentrations) in the presence of 1 mg L^−1^ iron(III) sulfate, iron(III) chlorate, or iron(III) chloride salts, (Table 3, entry 9). When using H_2_O_2_ (optimal concentration of 40 mg L^−1^), DIM completely disappeared after 15 min of irradiation, whereas MPA required 50 min of reaction time for complete disappearance. The authors observed an increase in reaction rate when **Fe_2_(ClO_4_)_3_** was used as the Fe^3+^ source, ascribed to inhibitory sulfate complexation effects on metal ion reactivity and chloride (Cl^−^) side reactivity with HO^•^. In addition, the activation of K_2_S_2_O_8_ by Fe^3+^ in combination with UV light was also examined, and it was found that the **UV/Fe^3+^/K_2_S_2_O8** system showed higher reactivity. This can be likely attributed to the high photolytic degradation capacity of K_2_S_2_O_8_, generating SO_4_^•−^ radicals, which are more reactive than HO^•^. The use of K_2_S_2_O_8_ was also beneficial for dissolved organic carbon (DOC) reduction/removal, reaching 95% DOC reduction using **Fe_2_(ClO_4_)_3_** and S_2_O_8_^2−^ oxidant (DIM in 6 h; MPA in 3 h). The authors also observed a toxicity decrease when analyzing the photoproducts through a Microtox test. Both DIM and MPA degradation reactions, which employed **Fe_2_(ClO_4_)_3_** and H_2_O_2_ as oxidants, showed a more significant decrease in the toxicity of the photoproducts. This decrease was attributed to higher toxicity caused by the S_2_O_8_^2−^ oxidant and **Fe_2_(SO_4_)_3_** catalyst.

Poulios [94] conducted a study on the homogenous aqueous photo-Fenton degradation of imidacloprid (IMI), using ferrioxalate (**Fe_2_(oxal)_3_**) as a catalyst and H_2_O_2_ with a 200 mg L^−1^ concentration. The study compared this homogenous procedure with other heterogeneous metal oxide photo-assisted systems (Table 3, entry 10). The photo-Fenton process has the theoretical advantage of being able to utilize sunlight, thereby avoiding high UV lamp costs. Low pH values are necessary to prevent iron precipitation at higher pH levels, and to facilitate its removal after treatment. Under UV-A irradiation, IMI abatement was more efficient, reaching 90% deg. and 80% DOC reduction in 90 and 120 min, respectively. Visible irradiation led to 85% deg. and 70% DOC reduction within 120 min, with a consequent experimentally observed toxicity decrease of the photoproducts. Direct comparison of photo-Fenton and **TiO_2_**-mediated processes allowed the authors to observe that, under UV-A irradiation and pH = 3.2, the **Fe^3^**^+^/H_2_O_2_/UV-A system could degrade IMI in 65%, while the **TiO_2_**/UV-A system only reached 40% deg., both within 30 min. A hybrid system combining **Fe^3+^/TiO_2_/**H_2_O_2_/UV-A was also analyzed and demonstrated great potential by achieving > 80% IMI degradation.

This approach was later employed by Abramović [95], who prepared **Fe^3+^/TiO_2_** hybrid photocatalysts with various Fe contents to promote the degradation of thiacloprid (THI) under UV irradiation with a 125 W high-pressure Hg lamp (Table 3, entry 11). Of the photocatalysts prepared (having 1.0, 7.2, and 13.9 *w*/*w* % of Fe, relative to Ti), the one with 7.2% *w*/*w* Fe showed the best catalytic properties (1.67 gL^−1^ concentration), achieving full THI degradation after only 25 min irradiation in the presence of [H_2_O_2_] = 1.44 g L^−1^. By comparison, pristine **TiO_2_** and **TiO_2_/H_2_O_2_** systems showed an 84% and 83% degradation rate, respectively. This system also allowed a TOC reduction of 90% after 240 min irradiation and reutilization for 3 cycles with negligible activity loss. The same group [96] extended their studies into implementing a metal free aqueous photodegradation catalytic system, using a 125 W high-pressure Hg lamp for irradiation in the presence of H_2_O_2_ under a concentration of 4400 mg L^−1^ at pH = 2.8 and 25 °C (Table 3, entry 12). The authors were able to degrade THI in 97% after 120 min irradiation, showing a first-order rate constant k = 0.027 min^−1^ at a H_2_O_2_/THI molar ratio of 220 and pH 2.8. The authors further observed that, after 35 h of irradiation, 17% of organic carbon remained nondegraded.

**Figure 8:**
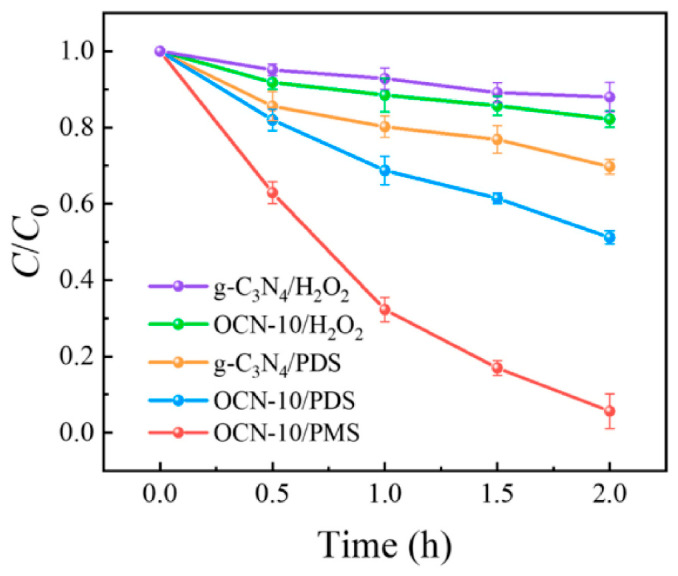
Effect of different oxidants on the IMI degradation under visible light. Adapted with permission from ref. [97]. Copyright 2022 Elsevier B.V., Amsterdam, The Netherlands.

Other researchers [97] studied the impact of the oxidant on the metal-free photocatalyzed oxidation of imidacloprid (IMI). Peroxymonosulfate (PMS), potassium peroxodisulfate (PDS), and hydrogen peroxide were compared as oxidizing agents, which were activated by oxygen-doped graphitic carbon nitride (**OCN**) (Figure 8). Under a PMS/IMI ratio of 1000:1 and irradiation using a 500 W Vis Xe lamp, 95% of IMI was degraded in 2.0 h, with a first-order rate constant of k = 0.025 min^−1^. This was ~30 times higher than when the pristine **g-C_3_N_4_**/PMS was used as a catalyst (Table 3, entry 13). The PMS oxidation on electron-deficient carbon atoms and holes, the PMS reduction around electron-rich O atoms and photogenerated electrons, and the multiple reactions of superoxide radical were the sources of the main active species singlet oxygen. Additionally, the catalyst was able to be reused up to five times without losing its activity.

Meenakshi [98] evaluated the efficacy of a metal-free photocatalyst, consisting of graphitic carbon nitride (**g-C_3_N_4_**) incorporated into chitosan (**CS**) for degrading chlorpyrifos (CPF), using a 300 W Xe lamp as an irradiation source, but without the use of an oxidant (Table 3, entry 14). The use of **CS/g-C_3_N_4_** to degrade the pesticide demonstrated high efficiency of about 85% after 50 min irradiation, with a first-order rate constant of k = 0.084 min^−1^. The authors identified HO^•^ and h^+^ as primary sources of active species and attributed the photocatalytic performance to an efficient separation of electron-hole pairs, with chitosan functioning as the charge separation carrier, enabling the catalyst to be reused for at least 5 cycles without loss of catalytic activity.

### 2.3. Using Tetrapyrrole-Based Macrocycle Photocatalysts

**Figure 9:**
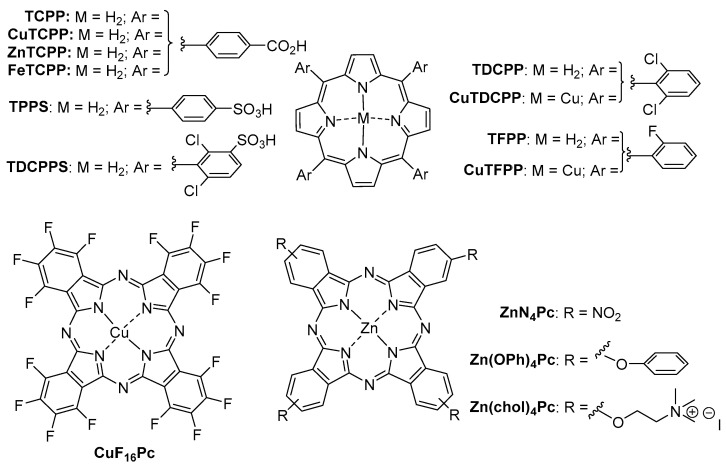
Structures of tetrapyrrolic macrocycles used as photocatalysts in the degradation of pesticides.

Another approach in the exploitation of the high destructive potential of ROS in pollutant degradation regards the use of tetrapyrrolic macrocycles (TPM) as photocatalysts in the degradation of pesticides, though to a much lesser extent (Figure 9).

Table 4 summarizes the existing literature on processes and reaction conditions to promote the photodegradation of pesticides, using tetrapyrrole-based macrocycles as photocatalysts.

Chovelon and colleagues [99] were the first to report the application of a tetrapyrrolic macrocycle, specifically tetra(4 carboxyphenyl)porphyrin, coordinating a set of metals (Fe(III), Cu(II), Zn(II), and metal-free **MTCPP**, Figure 9) immobilized on **TiO_2_** as photocatalysts for the photodegradation of a pesticide (atrazine-ATR). The reaction was performed in aqueous conditions, irradiated with a UV 250 W Hg lamp (Table 4, entry 1). The authors observed that the use of hydrogen peroxide as a green oxidant favored degradation. The copper (II)-porphyrin (**CuTCPP**) and H_2_O_2_ at 0.015–0.05 mol L^−1^ exhibited the highest photocatalytic activity among the photosensitizers tested, reaching 82% ATR photodegradation, after 1 h irradiation. Thirteen photoproducts were identified and characterized by HPLC-MS, with HO^•^ being identified as the main ROS.

Liu and collaborators [100] applied a nanoscale porphyrinic metal−organic framework (MOF) based on 5,10,15,20-tetrakis(4-carboxyphenyl)porphyrin (**TCPP**, Figure 9) for the detection (through fluorescence) and photodegradation of nitenpyram (NIT) (Table 4, entry 2). The authors evaluated this photocatalytic process under dark and irradiation conditions using a laser at 660 nm (300 mW cm^−2^ for 20 min) and analyzed the photodegradation rate by HPLC. Up to 95% photodegradation was observed through ROS formation (mainly ^1^O_2_ and ^•^OH).

Pereira and collaborators [101] examined the potential of water-soluble porphyrins **TPPS** and **TDCPPS** (depicted in Figure 9) as photocatalysts for degrading atrazine (ATR) and ametryn (AME) pesticides (Table 4, entry 3). The studies were conducted at various pH values (2.1, 5.8, 7.2, and 12.0) and the photoproducts were analyzed by GC-FID and HPLC-UV. ATR showed 30% photodegradation and AME showed 63% photodegradation after 120 h of irradiation with a low-pressure monochromatic lamp (λ = 350 nm). Degradation mechanism was linked to the formation of O_2_^−•^ and HO^•^ species.

The same group [102] promoted the immobilization of halogenated porphyrins (**TDCPP**, **TFPP**, Figure 9) and their copper(II) complexes **CuTDCPP** and **CuTFPP** (Figure 9) onto MCM-41 and assessed the photodegradation of 2,4,6-trimethylphenol (2,4,6-TMP), fenamiphos (FEN) and diuron (DIU) (Table 4, entry 4). The photocatalyst **TFPP**@MCM-41 was the most effective one for the degradation of model pesticide 2,4,6-TMP, reaching 80% degradation after 5 h irradiation using UV light. This photosensitizer was then further tested in the degradation of FEN and DIU, reaching 60% degradation of FEN after 5 h irradiation. Additionally, the photodegradation products were characterized using LC-MS and the heterogeneous photocatalysts were reused without loss on photocatalytic activity in different recycling cycles. The same research group [103] also reported the immobilization of **TFPP** on Na-Y zeolite for the photodegradation of 2,3,5-trimethylphenol (2,3,5-TMP) and mecoprop (MEC) (Table 4, entry 5). This catalytic system exhibited a photodegradation activity of more than 75% as determined by HPLC, following a 3 h exposure to 125 W medium-pressure mercury lamps.

In regards to the use of phthalocyanines as photocatalysts, Serra and collaborators [104] conducted a study where they covalently immobilized **CuF_16_Pc** (Figure 9) within hexagonal mesoporous silica. Then, they assessed the photocatalytic activity of the system on the degradation of 2,4-dichlorophenoxyacetic acid (2,4-DCPA) (Table 4, entry 6). The catalysts exhibited a high photocatalytic activity, exceeding 90% degradation after only 30 min irradiation with a 125 W mercury vapor lamp. However, the study identified a decrease in photocatalytic activity (from 90% to 60%) on 2,4-DCPA degradation after six photocatalytic cycles.

**Figure 10:**
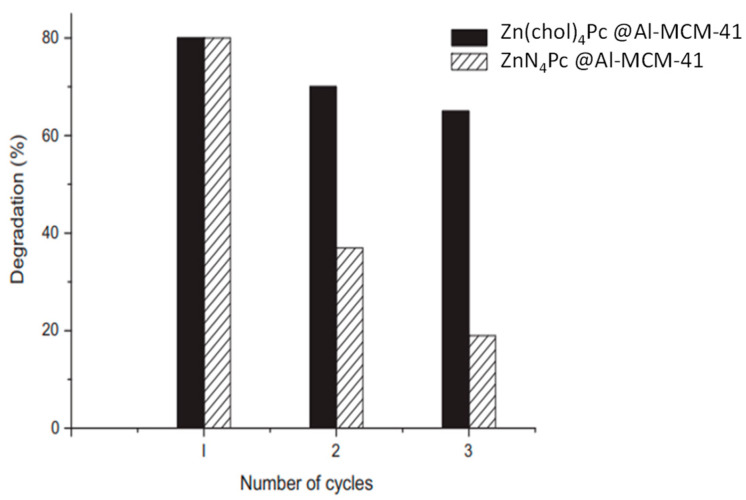
Photocatalytic cycles of **ZnTNPc**@Al-MCM-41 and **Zn(chol)_4_Pc**@Al-MCM-41 as catalysts on photodegradation of fenamiphos. Adapted with permission from ref. [105]. Copyright 2012 Elsevier B.V., Amsterdam, The Netherlands.

Pereira and Azenha [105] conducted a study on the photodegradation of pesticides fenamiphos (FEN) and pentachlorophenol (PCP) using a set of Zn(II) phthalocyanines, namely **ZnN_4_Pc**, **Zn(OPh)_4_Pc**, and **Zn(chol)_4_Pc** (Figure 9). The phthalocyanines were immobilized into Al-MCM-41 (Table 4, entry 7), which served as inorganic support. According to the authors, **ZnN_4_Pc** and **Zn(chol)_4_Pc** catalysts were the most active photocatalytic systems using three 125 W mercury lamps during 180 min. The authors identified the FEN photoproducts by LC-MS and studied the stability and reusability of the photocatalysts (Figure 10). The authors observed higher stability of the **Zn(chol)_4_Pc** system (in black) when compared to the **ZnN_4_Pc** catalysts (in grey) after 3 cycles of reutilization.

## 3. Conclusions

From the literature analysis conducted, one can conclude that the scientific community has dedicated its efforts towards the search for more environmentally friendly and effective catalytic processes for pesticide degradation. The previously stated findings indicate that many authors consider significant red shifts in the absorption edges of photocatalysts, leading to a narrowing of the band gap and an expanded light-harvesting window, along with reduced recombination of charge carriers, as key characteristics for designing an ideal photocatalyst.

Considering the aforementioned examples, we emphasize Madras’ work [79], who transitioned from traditional TiO_2_-based semiconducting photocatalysts to a CuS semiconductor with the objective of increasing the catalysts’ bandgap and preventing charge recombination. The semiconductor was subsequently doped with sodium thiosulfate. After being exposed to a 400 W metal halide lamp for five hours, this study demonstrated remarkable results, including the highly efficient breakdown of 4-chlorophenol into smaller components, resulting in a 40% reduction in TOC. The reusability of the catalyst for up to 10 cycles was also impressive. The same objective was pursued by Kalantary [89] by utilizing reduced graphene oxide (rGO) as a carbon source, together with magnetic Fe_3_O_4_ and ZnO, to create a ternary photocatalyst that effectively decomposed metalaxyl, attaining 92% degradation. The catalyst was reused for 5 cycles, with negligible loss of activity. Significantly, a 51% decrease in TOC was attained after 2 h of irradiation, while detecting the presence of four photoproducts. It was observed that there was a decrease in toxicity compared to the pesticide. Pereira and Azenha’s study [105] also provides an important example of using Zn(II) phthalocyanines immobilized into inorganic Al-MCM-4 as photocatalysts for the photodegradation of pesticides fenamiphos and pentachlorophenol. The photosensitizers exhibited high activity, stability, and reusability.

These examples suggest that further efforts should be made in designing and scaling-up efficient photocatalysts to improve their longer wavelength absorption and reduce charge carrier recombination through appropriate semiconductor doping and semiconductor combination. Tetrapyrrolic macrocycles (TPMs) are an ideal component in creating these desired photocatalysts. While no definitive examples have been reported using transition metal complexes (TPMs) combined with semiconductors, this may be a promising and significant approach for improving photocatalysts. TPMs are easily modulated and functional, enabling them to effectively induce higher levels of activity and stability, while also forming stable links to a variety of supports, including semiconductors. This has the added benefit of preventing catalyst leaching during reuse.

Further attention should be given to the light source when designing a photocatalytic system, preferably utilizing solar or visible energy. Additionally, in designing oxidative chemical systems, preference should be given to environmentally benign oxidants. Another crucial challenge that demands attention is the achievement of complete photodegradation of pesticides. When partial mineralization occurs, it is crucial to evaluate the generated byproducts to ensure reduced toxicity and environmental persistence. The majority of studies reported here support this approach. In stark contrast to the promising laboratory findings, a meaningful correlation between them and the real-world pilot-scale applications, specifically those utilizing solar energy, requires further investigation. This includes evaluating degradation efficiency, photoproducts formed, degrees of mineralization, and toxicity.

## Figures and Tables

**Table 1:** Categories of pesticides studied in catalyzed photodegradation, discussed in this review.

Category	Structures
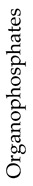	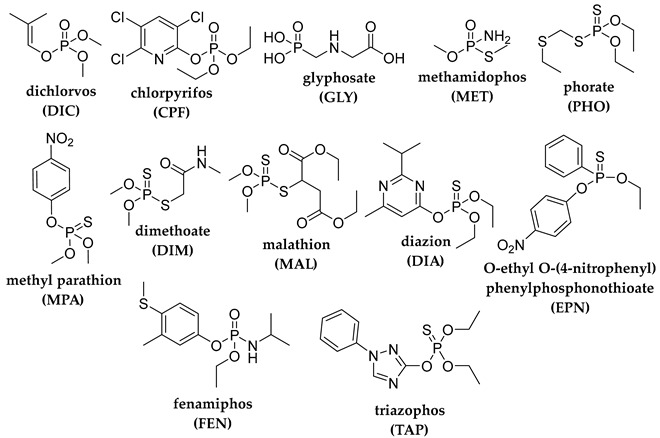
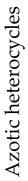	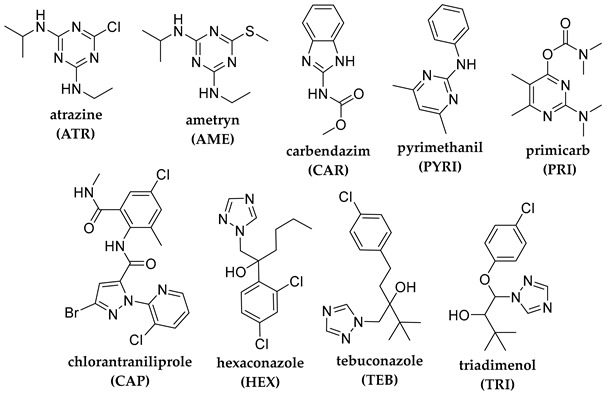
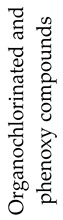	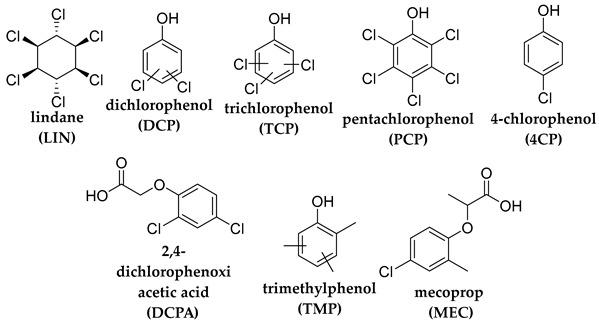
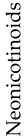	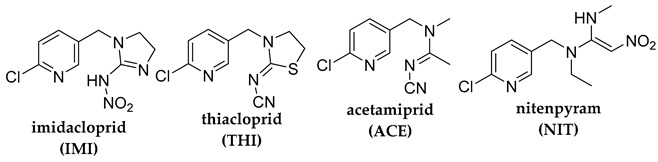
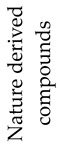	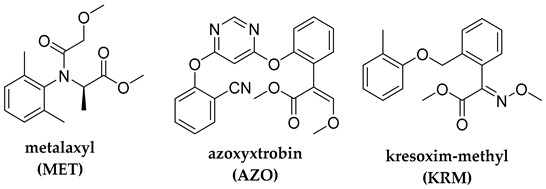
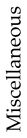	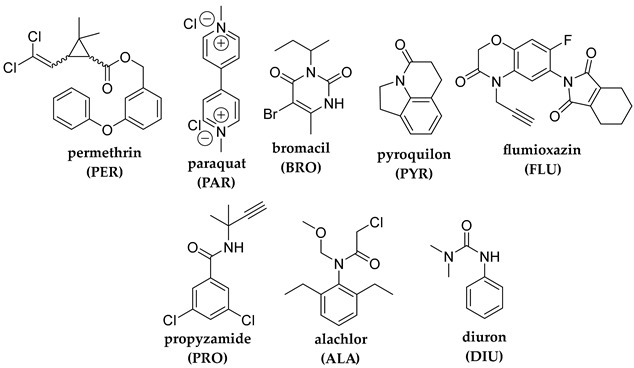

**Table 2:** Selection of examples of photochemical degradation of pesticides using metal oxide semiconductors (for pesticide names/structures, see Figure 1).

Entry/ Ref.	Substrates and Catalysts	Operational Features	Results and Comments
1 [73]	[PCP] = 12 mg L^−1^ [**TiO_2_**] = 2000 mg L^−1^	- Irradiation: Solar simulator; 310 < λ < 830 nm; intensity= 3.3 × 10^−5^ E/min - Matrix: Deionized water (DW); pH = 3; T = 45–50 °C	- 99% deg. PCP after 1.5 h
2 [74]	[PER] = 17,000 mg L^−1^ [**T-805**] = 100 mg L^−1^ [**TiO_2_** (P-25)] = 100 mg L^−1^	- Irradiation: UV light; λ > 330 nm; solar sunlight; 300 < λ < 460 nm; intensity= 6 mW cm^−2^ - Matrix: hexane-water mixture; pH = 5.7; T = not adjusted	- 90% deg. PER under UV light, after 20 h; 80% deg. PER under solar sunlight, after 10 h, both for **T-805** - Kinetics: k_1_ = 0.002 min^−1^ for **T-805** - Mineralization: 5% CO_2_ measured for **T-805**, after 24 h
3 [75]	[4-CP], [2,4-DCP], [2,4,6-TCP], [2,3,6-TCP], [2,3,5-TCP], [PCP], [LIN] = 4.6 to 7.5 mg L^−1^ [**TiO_2_ disks**] = NR	- Irradiation: UV lamp; 260 < λ < 400 nm; intensity = 0.6 mW cm^−2^ - Matrix: DW; acetone (for LIN); pH = 7; T = 25 °C	- ≥90% deg. 4-CP, 2,4-DCP, 2,3,5-TCP, and PCP; 86–89% deg. 2,4,6-TCP and 2,3,6-TCP; 63% deg. LIN - Kinetics: k_1_ = 0.032 min^−1^ (4-CP), 0.067 min^−1^ (2,4-DCP), 0.017 min^−1^ (2,4,6-TCP), 0.020 min^−1^ (2,3,6-TCP), 0.090 min^−1^ (2,3,5-TCP), 0.025 min^−1^ (PCP), 0.005 min^−1^ (LIN).
4 [76]	[ATR] = 25 mg L^−1^ [**TiO_2_**] = 1000 mg L^−1^	- Irradiation: solar simulator with Xe lamps; λ = 290 nm; intensity = 50 to 90 mW cm^−2^ - Matrix: DW; pH = 5; T = 70 °C	- ~90% deg. for ATR after 60 min - 90% DOC reduction
5 [77]	[CAR] = 10 mg L^−1^ [**TiO_2_**] = 70 mg L^−1^	- Irradiation: 250 W Hg lamp; 280 < λ < 400 nm; intensity = 90 mW cm^−2^ - Matrix: DW; pH = 6.73; T = 25 °C	- 90% deg. CAR - Kinetics: k_1_ = 0.030 min^−1^
6 [78]	[AZO] = [KRM] = [HEX]= [TEB] = [TRI] = [PYRI] = [PRO]= [PRO] = 0.5 mg L^−1^ [**ZnO**]= 150 mg L^−1^	Irradiation: natural sunlight; 400 nm < λ < 700 nm Matrix: greenhouse irrigation water pH = 8–8.4; T = 25 °C Na_2_S_2_O_8_: 100 mg L^−1^	- >99% deg. in all cases (1–2 h) - Kinetics: decreasing k_1_ = 1.4727, 0.7370, 0.7019, 0.6014, 0.5862, 0.5475, 0.4762, and 0.3885 min^−1^ for PRI, PYRI, PRO, AZO, KRM, TEB, HEX, and TRI, respectively.
7 [79]	[4-CP]= 100 mg L^−1^ [**CuS-ST**] = [**CuS-TA**] = [**CuS-TU**] = 50 mg L^−1^	Irradiation: 400 W metal halide lamp; 400 nm < λ < 700 nm Matrix: DW pH = 8–8.4; T ~25 °C	- >62%, 40% and 25% deg. for **CuS-TU**, **CuS-TA** and **CuS-ST**, respectively. - Kinetics: k = 0.003, 0.002 and 0.0017 min^−1^ for **CuS-TU**, **CuS-TA**, and **CuS-ST**, respectively. - 40% TOC decrease after 5 h of irradiation using CuS-TU - Reutilization: 10 cycles

**Table 3:** Selection of pesticide degradation processes using hybrid photocatalysts (for pesticide names/structures, see Figure 1).

Entry/Ref	Substrates and Catalysts	Operational Features	Results and Comments
1 [85]	[CAP]_0_ = 0.1 mg/L [**TiO_2_**] = 300 mg L^−1^ [**ZnO**] = 200 mg L^−1^ [**Zn_2_TiO_4_**] = 200 mg L^−1^	- Irradiation: 8 W medium pressure Hg lamps; 300 < λ < 460 nm; intensity = 10 mW cm^−2^ - Matrix: deionized water (DW); pH = 7 (for **ZnO** and Zn_2_TiO), pH = 6 (for **TiO_2_**); T = 25 °C	- 99%, 98%, and 80% deg. CAP for **ZnO**, **TiO_2_**, and **Zn_2_TiO_4_**, respectively, after 1 h (in the presence of Na_2_S_2_O_8_) - Kinetics: k = 0.008, 0.013, and 0.002 min^−1^ for **TiO_2_**, **ZnO**, and **Zn_2_TiO_4_**, respectively - 7 PPs detected - ~90% DOC reduction for **TiO_2_**, **ZnO**; 22% for **Zn_2_TiO_4_**
2 [86]	[CPF]_0_ = 10 mg/L [**CuS_10_/Bi_2_O_2_CO_3_(U_5_K_1_)**] = 250 mg L^−1^	- Irradiation: 150 W Xe lamp; 200 < λ < 800 nm; intensity: NR - Matrix: DW; pH = 4; T = 25 °C	- 95% deg. CPF after 3 h - Kinetics: k = 0.031 min^−1^ - 8 PPs detected - ~90% TOC reduction, after 3 h - Reutilization: 5 reuse cycles without loss of activity
3 [87]	[PAR] = 20 mg L^−1^ [**g-C_3_N_4_/Bi_2_O_2_CO_3_/ CoFe_2_O_4_**] = 500 mg L^−1^	- Irradiation: 800 W Xe lamp; 200 < λ < 800 nm; intensity: NR and natural sunlight - Matrix: DW; pH = 7; T = 30 °C	- 99% deg. PAR under visible radiation after 90 min, and 92% under solar light after 120 min - Kinetics: k = 0.069 min^−1^ under Xe lamp; k = 0.060 min^−1^ under sunlight - 57% - of TOC reduction in 3 h
4 [88]	[GLY] = 50 mg L^−1^ [**g-C_3_N_4_/BiOI/Bi_2_MoO_6_**] = 250 mg L^−1^	- Irradiation: tungsten halogen lamp; λ: NR; intensity: NR - Matrix: DW; pH = not adjusted; T = 25 °C	- 94% deg. GLY, after 90 min - Kinetics: k = 0.018 min^−1^ - ~83% TOC reduction, after 4.5 h - Reutilization: 5 reuse cycles with 5% loss of activity
5 [89]	[MET]_0_ = 30 mg/L [**(rGO)/Fe_3_O_4_/ZnO**] = 500 mg L^−1^	- Irradiation: 5 Vis-LED lamps; λ = 450 nm; intensity = 160 W m^−2^ - Matrix: DW; pH = 7; T = 25 °C	- 92% deg. MET, after 2 h - Kinetics: k = 0.023 min^−1^ - 4 PPs detected - ~51% TOC reduction, after 2 h - Reutilization: 5 reuse cycles with 5% loss of activity - Toxicity: PPs less toxic than MET (experimentally observed)
6 [90]	[PYR] = [IMI] = 10 mg L^−1^ [**g-C_3_N_4_/Ag_3_PO_4_@Q [7]**] = 400 mg L^−1^	- Irradiation: 35 W LED lamp; λ: NR; light intensity: NR - Matrix: DW; pH = not adjusted; T = 25 °C	- 94% and 97% deg. PYR and IMI, respectively, after 5 h - Kinetics: k = 0.009 and 0.012 min^−1^ for PYR and IMI, respectively - Reutilization: 5 reuse cycles; 20% and 11% activity drop from PYR and IMI deg.
7 [91]	[MET] = [MAL] = [DIA] = [PHO] = [EPN] = 8 mg L^−1^ [**Fe(SO_4_)**] = 7.6 mg L^−1^ [**Fe^0^**]= 830 mg L^−1^	- Irradiation: 100 W medium pressure Hg lamp; 253 < λ < 578 nm; intensity: 2.3 W m^−2^ - Matrix: DW; pH = 7; T = 25 °C - [H_2_O_2_] = 20 mg L^−1^	- all degraded after 150 min (homogeneous system) - Kinetics with Fe^2+^: k = 0.021, 0.020, 0.013, 0.012, and 0.011 min^−1^ for MET, PHO, EPN, MAL and DIA, respectively
8 [92]	[ACE] = 0.100 mg L^−1^ **Iron(II) sulfate** [FeSO_4_] = 3 mg L^−1^	- Irradiation: three 30 W UVC low pressure lamps (LPL); λ = ~254 nm; or three 30 W UVC LED; λ = ~256 nm; intensity = 20 W m^−2^ for both - Matrix: DW and synthetic wastewater; pH = 2.8 and 8.2; T =20 °C - [H_2_O_2_] = 12 mg L^−1^	- 100% deg. ACE after 7 min (at pH = 2.8, synthetic wastewater) with LPL system - 100% deg. ACE after 20 min (at pH = 8.2, synthetic wastewater) with LPL system
9 [93]	[DIM] = [MPA] = 10 mg L^−1^ [**Fe_2_(SO_4_)_3_**] = [**Fe_2_(ClO_4_)_3_**] = [**FeCl_3_**] = 1 mg L^−1^	- Irradiation: 125 W high pressure Hg lamp - λ > 290 nm; 30 W m^−2^ - Matrix: DW; pH = 2.9; T =30–35 °C - [H_2_O_2_] = 40 mg L^−1^ - [S_2_O_8_2^−^] = ~300 mg L^−1^	- 100% deg. after 15 min (DIM) and 50 min (MPA); (homogeneous system using H_2_O_2_ oxidant ) - 100% deg. after 10 min (DIM) and 20 min (MPA); (homogeneous system using S_2_O_8_^2−^ oxidant) - 95% DOC reduction using **Fe_2_(ClO_4_)_3_** and S_2_O_8_^2−^ oxidant (DIM in 6 h; MPA in 3 h) - 3 PPs detected for DIM and 8 PPs for MPA - greater toxicity decrease when using **Fe_2_(ClO_4_)_3_**
10 [94]	[IMI] = 20 mg L^−1^ **Iron(III) oxalate** [**Fe_2_(oxal)_3_**] = 7 mg L^−1^	- Irradiation: UV-A; 340 < λ < 400 nm; light intensity: 1.0 × 10^−4^ E min^−1^ or 9 W Vis lamp; 400 < λ < 550 nm; intensity: 6.0 × 10^−5^ E min^−1^ - Matrix: DW; pH = 3.2; T =25 °C - [H_2_O_2_] = 200 mg L^−1^	- 90% deg. IMI under UV-A; 85% deg. IMI under Vis irradiation, both in 120 min - 80% DOC reduction under UV-A in 90 min; 70% DOC reduction under Vis, in 120 min - 4 PPs detected - toxicity decrease
11 [95]	[THI] = 20 mg L^−1^ 7.2% *w*/*w* Fe (denoted as **7.2Fe/TiO_2_**) [**7.2Fe/TiO_2_**] = 1670 mg L^−1^	- Irradiation: 125 W high-pressure Hg lamp; λ > 290 nm; light intensity: 1.5 × 10^−5^ E min^−1^ - Matrix: DW; pH = 2.8; T =25 °C - [H_2_O_2_] = 1440 mg L^−1^	- 100% deg. THI after 25 min - >90% TOC decrease after 240 min - Reutilization: 3 reuse cycles
12 [96]	[**THI**] = 20 mg L^−1^ [**7.2Fe/TiO_2_**] = 1670 mg L^−1^	- Irradiation: 125 W high-pressure Hg lamp; λ > 290 nm; light intensity: 2.54 × 10^−7^ E s^−1^ - pH = 2.8; T =25 °C - [H_2_O_2_] =4400 mg L^−1^	- 97% deg. THI after 120 min - Kinetics: k = 0.027 min^−1^ - 83% TOC reduction in 35 h
13 [97]	[IMI] = 3 mg L^−1^ [**OCN**] = 500 mg L^−1^	- Irradiation: 500 W Vis Xe lamp; 420 < λ < 800 nm; light intensity: NR - pH = 4.2; T =25 °C - [PMS] = 3 g L^−1^	- 95% deg. IMI after 2 h - Kinetics: k = 0.025 min^−1^ - Reutilization: 5 reuse cycles - 11 PPs detected
14 [98]	[CPF] = 50 mg L^−1^ [**CS/g-C_3_N_4_**] = 2 mg L^−1^	- Irradiation: 300 W Xe lamp; 200 < λ < 800 nm; light intensity: NR - pH = 5.3; T =25 °C	- 85% deg. CPF after 50 min - Kinetics: k = 0.084 min^−1^ - Reutilization: 5 reuse cycles

**Table 4:** Photodegradation of pesticides using tetrapyrrolic macrocyclic derivatives as photocatalysts (for pesticide names/structures, see Figure 1).

Entry/Ref	Substrates and Catalysts	Operational Features	Results and Comments
1 [99]	[ATR] = 10 mg L^−1^ [**TCPP**] = [**CuTCPP**] = [**ZnTCPP**] = [**FeTCPP**] = 1 mg L^−1^	- Irradiation: UV 250 W Hg lamp (λ > 290 nm) - Matrix: distilled water; pH = 5.5; T = 20 °C - [H_2_O_2_] = 0.015–0.05 mol L^−1^	- >82% ATR in 1 h of irradiation - 13 PPs detected
2 [100]	[NIT] = 10 mg L^−1^ [**TCPP**]: 10 mg L^−1^	- Irradiation: laser light (λ = 660 nm) intensity = 300 mW cm^−2^ - Matrix: PBS buffer; pH 7.4; T = NR	- >95% NIT in 20 min of irradiation
3 [101]	[ATR] =[AME] = 1 mg L^−1^ [**TPPS**] = 4 mg L^−1^ [**TDCPPS**] = 5 mg L^−1^	- Irradiation: low-pressure mercury monochromatic lamp (λ = 350 nm) - Matrix: water; pH = 2.1, 7.2 and 12.0; T = NR	- >30% (ATR) and 63% (AME) in 120 h of illumination - 2 PPs detected
4 [102]	[2,4,6-TMP] = 15 mg L^−1^; [FEN] = 33 mg L^−1^ [DIU] = 26 mg L^−1^ [**TDCPP**] = [**CuTDCPP**] = [**TFPP**] = [**CuTFPP**] = 500 mg L^−1^	- Irradiation: six 15 W UV lamps; range 300–460 nm (λmax = 366 nm); - Matrix: water; pH = 6.0; T = 25 °C	- 80% deg. 2,4,6-TMP in 5 h of irradiation (with **TFPP**) - 60% deg. FEN in 5 h of irradiation (with **TFPP**) - Kinetics: 0.0025 min^−1^ for FEN (with **TFPP**) - PPs: 4 PPs detected (DIU) and 2 PPs detected (FEN)
5 [103]	[2,3,5- TMP] = 68 mg L^−1^; [MEC] = 107 mg L^−1^ [**TFPP**] = 200 mg L^−1^	- Irradiation: three 125 W medium-pressure mercury lamps - Matrix: water; pH = 6.0; T = 23 °C	- >75% in 3 h of irradiation (for both pesticides) - 2 PPs detected (MEC)
6 [104]	[2,4-DCPA] = 10 mg L^−1^ [**CuF_16_Pc**] = 500 mg L^−1^	- Irradiation: 125 W mercury vapor lamp with an irradiance of 970 μW/cm^2^ - Matrix: water (H_2_O_2_; 1.2 wt%); T = 25 °C	- >90% in 30 min of irradiation - Reutilization: photocatalytic activity reduction from 90% to 60% after six catalytic cycles
7 [105]	[FEN] = 30 mg L^−1^ and [PCP] = 3 mg L^−1^ [**ZnN_4_Pc**] = [**Zn(OPh)_4_Pc**] = [**Zn(chol)_4_Pc**] = 500 mg L^−1^	- Irradiation: three 125 W mercury lamps - Matrix: water; pH = 5.5; T = 25 °C	- >~90% in 180 min of irradiation - Kinetics: k = 0.003 min^−1^ (FEN) and 0.0011 min^−1^ (PCP) - 2 and 1 PPs detected for FEN and PCP, respectively - Reutilization: three cycles
